# Minimal-invasive approach for penetrating Crohn’s disease is not associated with increased complications

**DOI:** 10.1007/s00464-016-4871-4

**Published:** 2016-06-22

**Authors:** Ivan Kristo, Anton Stift, Stanislaus Argeny, Martina Mittlböck, Stefan Riss

**Affiliations:** 1 Department of Surgery, Medical University of Vienna, Währinger Gürtel 18-20, 1090 Vienna, Austria; 2 Center for Medical Statistics, Informatics and Intelligent Systems, Vienna, Austria

**Keywords:** Penetrating Crohn’s disease, Non-penetrating Crohn’s disease, Laparoscopic surgery, Crohn’s disease, Perforated Crohn’s disease

## Abstract

**Background:**

Laparoscopic surgery for penetrating Crohn’s disease (CD) still remains highly conflicting due to a lack of sufficient data. Therefore, the following large study was designed to compare postoperative outcomes after minimal-invasive resections for penetrating and non-penetrating CD.

**Methods:**

Consecutive patients, who underwent laparoscopic intestinal resection for symptomatic CD at a tertiary academic referral center, were included. Patients were divided according to perioperative findings in penetrating and non-penetrating type of disease. All clinical data were obtained from an institutional database and analyzed retrospectively.

**Results:**

Of 234 patients enrolled, 101 patients [females: *n* = 54 (53.5 %)] were operated on for non-penetrating CD and 133 patients [females: *n* = 50 (37.6 %)] for penetrating CD. Fistulas (*p* < 0.001), inflammatory mass (*p* < 0.001) and abscess formation (*p* < 0.001) were observed more frequently in the perforating group. Ileocolic resections were performed predominantly in both groups [perforating CD: *n* = 110 (82.7 %), non-perforating CD: *n* = 82 (81.2 %)], with more complex resections (>1 intestinal resection) found in perforating CD (*p* < 0.001). Conversion rates did not differ significantly. Notably, 30-day postoperative morbidity was comparable for both groups [perforating CD: *n* = 20 (15 %), non-perforating CD: *n* = 19 (18.8 %), *p* = 0.44]. Postoperative complication rates graded according to the Clavien–Dindo classification showed no difference too (*p* = 0.49).

**Conclusion:**

Laparoscopic surgery can be conducted safely in selected patients with penetrating CD without increasing the risk of postoperative complications. This finding needs to be implemented in future guidelines.

Crohn’s disease (CD) represents a challenging chronic inflammatory disorder, which is associated with an incidence of up to 20.2 per 100,000 in Northern America and Europe [1, 2]. The Vienna and Montreal classification systems subdivide disease behavior into either penetrating or stricturing or non-penetrating, non-stricturing phenotypes [3]. Greenstein et al. [4] described penetrating CD (PCD), characterized by acute perforation, chronic fistula or abscess formation, as a distinct aggressive type, which influences interval to reoperation. Recently, Niewadomski et al. [5] showed that penetrating disease, although treated by modern immunosuppressive medication, is still associated with a more severe course of disease. Given that typical features of penetrating disease are established risk factors for postoperative complications and adverse short-term outcomes in surgery, the question arises whether minimal-invasive surgery is an appropriate approach for PCD [6, 7].

Laparoscopic surgery is the treatment of choice for non-penetrating CD (NPCD) with well-proven benefits, such as less surgical trauma, earlier bowel function, less postoperative pain and shorter hospital stay when compared to open procedures [8, 9].

Nevertheless, technical difficulties such as the presence of inflammatory mass and bowel fistulas challenge the widespread use of the laparoscopic approach in complex CD resections. Additionally, Alves et al. [10] delineated that penetrating disease and preoperative immunosuppressive regimen increased the likelihood of conversion. Notably, current guidelines limit minimal-invasive techniques or even do not recommend laparoscopic resection as first-line treatment in penetrated CD due to insufficient data [11, 12].

Hereby, we present the largest study to date comparing outcomes of laparoscopic resection for penetrating and non-penetrating disease.

## Materials and methods

Consecutive patients, who underwent laparoscopic intestinal resections for symptomatic CD, were enrolled at a tertiary academic referral center between 1997 and 2012. Patients were divided according to perioperative findings and histological results in either penetrating or non-penetrating disease behavior. Penetrating phenotype was defined as acute free perforation, chronic perforation with fistula or abscess formation [4], whereas other operated patients were grouped as non-penetrating.

All minimal-invasive resections were conducted or supervised by experienced consultants, who are specialized in the treatment of CD. The surgical procedure was already described elsewhere in detail [13]. Briefly, after laparoscopic bowel mobilization exteriorization was performed through a 4–6 cm vertical periumbilical incision. Mesenteric vessel ligation, resection and anastomotic reconstruction were conducted extracorporeally. Conversion was defined as extension of planned incision (4–6 cm).

In 1997 at the beginning of the implementation of laparoscopic surgery for CD, a strict selection policy was performed. With increasing experience, even previous abdominal operations and complex cases were not regarded as contraindication for a minimal-invasive approach.

Demographic and relevant clinical data were extracted from an institutional database and individual chart review, respectively. The institutional review board approved the study protocol.

Next to baseline characteristics, we documented intraoperative findings and previous abdominal operations for CD. Steroid therapy was defined as the use of corticosteroids until the day before surgery. Those patients who received steroids preoperatively but stopped treatment at least 2 days before surgery were considered as “non steroids.” The exposure to azathioprine/6-mercaptopurine (AZA/6MP) before surgery was defined as the intake of AZA/6MP within 2 weeks preoperatively. The administration of anti-TNF antibody was documented within 1 week before operation. Resections were also divided into simple (1 intestinal resection) and complex (>1 intestinal resection).

Thirty-day morbidity was assessed by using the Clavien–Dindo Score [14]. In addition, complications were grouped into surgical and medical complications.

### Statistical analysis

Continuous data are shown as mean ± SD if normally distributed or as median and minimum–maximum otherwise. Categorical variables are described with absolute numbers and percentages and were tested by *χ* ^2^ test. In case of ordered categories, a trend version of the *χ* ^2^ test was used. Furthermore, an exact version of the *χ* ^2^ test was used if requirements for an asymptotic *χ* ^2^ test were not fulfilled. *T* tests were performed to compare means between two groups in case of normally distributed data and the Wilcoxon rank-sum test for skewed variables or in the presence of outliers. All *p* values were two-sided, and *p* ≤ 0.05 was considered statistically significant. All calculations were performed with SAS version 9.3 (SAS Institute Inc., Cary, NC, USA).

## Results

During the study period, 461 patients underwent surgery of CD. Two hundred and thirty-four (50.8 %) patients were operated on laparoscopically and were included for further analysis. A total of 133 patients (56.8 %) were assigned to the PCD group, and 101 patients (43.2 %) suffered from NPCD.

In the PCD group, a significantly higher number of male patients (*n* = 83, 62.4 %) were observed in contrast to patients with NPCD (*n* = 47, 46.5 %, *p* = 0.02). Further demographic data were comparable between both groups and are listed in Table 1.

**Table 1:** Demographic data of patients with penetrating and non-penetrating Crohn’s disease (CD)

	Penetrating Crohn’s disease	Non-penetrating Crohn’s disease	
Patients	133 (56.8)	101 (43.2)	
Age	32.6 (±11.6)	35.2 (±12.4)	NS
Sex			
Female	50 (37.6)	54 (53.5)	
Male	83 (62.4)	47 (46.5)	*p* = 0.02
BMI	22 (±4.5)	21.5 (±3.6)	NS
Age at CD diagnosis	23 (10–56)	25.5 (9–76)	NS
Smoking	68 (52.7)	50 (50.5)	NS
Azathioprine/6-mercaptopurine	21 (15.8)	23 (22.8)	NS
Corticosteroids	28 (21.1)	16 (15.8)	NS
Anti-TNF antibodies	0	3 (3)	NS
Previous CD surgery	16 (12)	27 (26.7)	*p* = 0.004

Intraoperative findings were significantly different between both groups in terms of the presence of inflammatory mass [PCD: *n* = 85 (63.9 %), NPCD: *n* = 14 (13.9 %), *p* < 0.0001], fistula [PCD: *n* = 118 (88.7 %), NPCD: *n* = 0, *p* < 0.0001] and abscess formation [PCD: *n* = 43 (32.3 %), NPCD: *n* = 0, *p* < 0.0001]. Additionally, patients with PCD underwent a higher number of complex [PCD: *n* = 55 (41.7 %), NPCD: *n* = 15 (14.9), *p* < 0.001] and colonic resections (*p* = 0.002, Table 2), which also was reflected by a longer postoperative hospital stay [PCD: 8 days (3–22), NPCD: 8 days (5–45), *p* = 0.033]. Operative time was comparable between groups [PCD: 135 (60–370) min, NPCD: 130 (50–360) min, *p* = 0.89).

**Table 2:** Comparison of type of resection and anastomosis between penetrating and non-penetrating Crohn’s disease

	Penetrating Crohn’s disease	Non-penetrating Crohn’s disease	
Type of resection			
Ileocolic	110 (82.7)	82 (81.2)	NS
Small bowel	14 (10.5)	14 (13.7)	NS
Colonic	34 (25.6)	11 (10.9)	*p* = 0.002
Rectal	1 (0.8)	0	NS
Anastomoses			
Stapled	89 (66.9)	71 (70.3)	NS
Handsewn	39 (29.3)	30 (29.7)	NS
Both	5 (3.8)	0	NS

Conversion rates tended to be higher when CD behaved penetrating [*n* = 22 (6.5 %) vs. *n* = 10, (9.9 %); *p* = 0.18], but failed to reach statistical significance. In the PCD group, conversion to open surgery was conducted because of inflammatory adhesions (*n* = 9, 40.9 %), large inflammatory mass (*n* = 7, 31.8 %), lack of anatomical clarity (*n* = 2, 9.1 %), severe fistula (*n* = 2, 9.1 %), bowel perforation (*n* = 1, 4.5 %) and bleeding (*n* = 1, 4.5 %). Reasons for conversions in the NPCD group were inflammatory adhesions (*n* = 7, 70 %), lack of anatomical clarity (*n* = 2, 20 %) and bleeding (*n* = 1, 10 %).

### Postoperative complications

There were no deaths after laparoscopic surgery for CD.

Surgical and medical complications did not differ between both groups (*p* = 0.39). In the PCD group, 20 (15 %) postoperative complications were observed [medical: *n* = 9 (6.8 %), surgical: *n* = 11 (8.3 %)]. Medical events were paralytic ileus (*n* = 4), fever of unknown origin (*n* = 3), pneumonia (*n* = 1) and cardiac arrhythmia (*n* = 1). Surgical complications were composed of anastomotic leakage (*n* = 4), wound infection (*n* = 4), postoperative bleeding (*n* = 2) and intra-abdominal abscess formation (*n* = 1).

In the NPCD group, 19 (18.8 %) postoperative events [medical complications: *n* = 12 (11.9 %), surgical complications: *n* = 7 (6.9 %)] were found. Medical complications included fever of unknown origin (*n* = 5), paralytic ileus (*n* = 4), urinary tract infection (*n* = 2) and pneumonia (*n* = 1), whereas anastomotic leakage (*n* = 3), mechanic bowel obstruction (*n* = 1), wound infection (*n* = 1), intra-abdominal abscess without leakage (*n* = 1) and bowel perforation due to drain arrosion (*n* = 1) accounted for surgical events, respectively.

Postoperative complications defined according to the Clavien–Dindo classification showed no significant difference between both groups (*p* = 0.49) and are further outlined in Table 3.

**Table 3:** Postoperative morbidity after laparoscopic resection in Crohn’s disease according to the Clavien–Dindo classification

	Penetrating Crohn’s disease	Non-penetrating Crohn’s disease	
No complications	113 (85)	82 (81.2)	NS
Grade I	8 (6)	4 (4)	
Grade II	4 (3)	9 (8.9)	
Grade IIIa	2 (1.5)	0	
Grade IIIb	6 (4.5)	5 (4.9)	
Grade IV	0	1 (1)	

## Discussion

To our knowledge, this is the largest study comparing the outcomes of laparoscopic surgery for penetrating versus non-penetrating CD. We could demonstrate that despite more aggressive clinical behavior, which was also reflected by more complex resections and a longer hospital stay, 30-day morbidity was not elevated in patients with perforated CD. Nevertheless, surgeons have to be aware that they are confronted with a trend to higher conversion rates in PCD.

Evidence of a distinct aggressive pattern in CD was already observed by Greenstein et al. [4] in 1988. Independent of anatomical localization, it was noticed that PCD retained its penetrating identity even after surgical resections. Importantly, phenotypic characteristics of PCD were also linked to postoperative complications and adverse short-term course.

Alves et al. [15] revealed abscess formation, recurrent CD episode, preoperative steroid use and poor nutritional status as risk factors for intra-abdominal septic complications after primary elective ileocolic resection. Kanazawa et al. [6] demonstrated that these findings were also responsible for recurrent interventions. They reviewed 550 patients from which 88.6 % underwent open and 11.4 % laparoscopic operations for primary and recurrent CD and confirmed PCD as a risk factor for postoperative septic complications. Furthermore, a high level of C-reactive protein, reflecting increased disease activity, a low level of preoperative hemoglobin and recurrent disease were highlighted as marker for an eventful postoperative course in laparoscopic CD surgery [13, 16, 17].

Deviation of planned operative approach is another important aspect in minimal-invasive surgery. Expert centers report conversion rates between 0 and 29 % in CD surgery [9]. Interestingly, Mino et al. [18] observed that fistulas and abscesses predicted conversion and suggested preoperative imaging as a tool to select patients without signs of PCD for laparoscopic interventions.

Consequently, current guidelines implemented those limited results about laparoscopic surgery in PCD. The European Crohn’s and Colitis Organisation (ECCO) stated that in complex cases or recurrent situations insufficient evidence exists to recommend laparoscopic surgery as technique of first choice [11]. In contrast, the American Society of Colon and Rectal Surgeons (ASCR) clarifies that a minimal-invasive approach in PCD is feasible if appropriate expertise is available although this recommendation is mainly based on a study with only 40 patients presenting PCD [12, 19, 20].

Previous reports about outcomes of surgery on PCD mainly focused on open resections or consisted of a small selected population of primary laparoscopic ileocolic resections [9, 21]. Thus, large comparative studies on minimal-invasive surgery in PCD are scarce.

Wu et al. [22] investigated laparoscopic-assisted ileocolic resections in CD and compared patients with abscess or phlegmon to recurrent disease and to patients that had an uncomplicated course of disease. Perioperative morbidity, conversion to an open procedure and operative times were comparable between minimal-invasive groups. Length of hospitalization was significantly shorter when laparoscopic approach was compared to a control group of 70 open resections. However, only 14 patients were classified to have an abscess or phlegmon, which limited the information on penetrating disease behavior.

Remarkably, Nguyen et al. [23] expanded information on outcomes of laparoscopic resections in CD. They retrospectively analyzed 335 minimal-invasive procedures by a single surgeon, which revealed a conversion rate of only 2 % and postoperative complications in 13 %. Though approximately one-third of the population was affected by PCD, results were not differentiated by type of disease behavior.

Interestingly, we noticed a longer postoperative hospital stay in the PCD cohort, which is concordant with a recent study that compared outcomes of PCD versus NPCD [24]. Bellolio et al. analyzed 293 patients with perforating CD, which were compared to 141 non-perforating phenotypes. Perforating phenotype was less likely to undergo laparoscopic procedure and developed more postoperative abscesses, but general morbidity was similar between groups. Nevertheless, results should be extrapolated with caution, because open and minimal-invasive techniques were mixed in both groups, and therefore information on laparoscopic outcomes in PCD should be interpreted carefully.

Few limitations of the study need to be addressed. Although the current analysis includes a high number of patients, the study was designed retrospectively; thus, selection bias cannot be ruled out. Our aim is to treat the majority of CD patients by using a minimal-invasive approach; however, patients with previous laparotomies in association with penetrating disease are still often managed by laparotomy. In contrast, those patients with a previous laparoscopic approach are initially attempted to be treated minimal invasive again even if a penetrating disease is expected. As a consequence, careful patient selection is crucial in order to achieve best outcome.

## Conclusion

In the present large series, we could clearly demonstrate that laparoscopic surgery is safe and feasible in perforating CD without an increased number of complications in comparison with non-perforating CD. This finding should be taken into account in future guidelines. Notably, laparoscopic surgery remains challenging in this group of patients; thus, careful patient selection and surgical experience is essential.
